# Children Use Statistics and Semantics in the Retreat from Overgeneralization

**DOI:** 10.1371/journal.pone.0110009

**Published:** 2014-10-15

**Authors:** Ryan P. Blything, Ben Ambridge, Elena V. M. Lieven

**Affiliations:** 1 Department of Psychological Sciences, University of Manchester, Manchester, United Kingdom; 2 Department of Psychological Sciences, University of Liverpool, Liverpool, Merseyside, United Kingdom; 3 ESRC International Centre for Language and Communicative Development (LuCiD), Department of Psychological Sciences, University of Manchester, Manchester, United Kingdom; Stony Brook University, United States of America

## Abstract

How do children learn to restrict their productivity and avoid ungrammatical utterances? The present study addresses this question by examining why some verbs are used with *un-* prefixation (e.g., *unwrap*) and others are not (e.g., *unsqueeze). Experiment 1 used a priming methodology to examine children's (3–4; 5–6) grammatical restrictions on verbal *un*- prefixation. To elicit production of *un*-prefixed verbs, test trials were preceded by a prime sentence, which described reversal actions with grammatical *un*- prefixed verbs (e.g., *Marge folded her arms and then she unfolded them*). Children then completed target sentences by describing cartoon reversal actions corresponding to (potentially) *un*- prefixed verbs. The younger age-group's production probability of verbs in *un-* form was negatively related to the frequency of the target verb in bare form (e.g., *squeez/e/ed/es/ing*), while the production probability of verbs in *un-* form for both age groups was negatively predicted by the frequency of synonyms to a verb's *un*- form (e.g., release/*unsqueeze). In Experiment 2, the same children rated the grammaticality of all verbs in *un-* form. The older age-group's grammaticality judgments were (a) positively predicted by the extent to which each verb was semantically consistent with a semantic “cryptotype” of meanings - where “cryptotype” refers to a covert category of overlapping, probabilistic meanings that are difficult to access - hypothesised to be shared by verbs which take *un-*, and (b) negatively predicted by the frequency of synonyms to a verb's *un*- form. Taken together, these experiments demonstrate that children as young as 4;0 employ pre-emption and entrenchment to restrict generalizations, and that use of a semantic cryptotype to guide judgments of overgeneralizations is also evident by age 6;0. Thus, even early developmental accounts of children's restriction of productivity must encompass a mechanism in which a verb's semantic and statistical properties interact.

## Introduction

An essential component of language acquisition is a speaker's ability to move beyond the linguistic input and use words in novel ways. For example, when verbs are observed in both the intransitive and transitive construction (e.g., *The ball bounced* → *The man bounced the ball*), a speaker may form an abstract linguistic generalization (e.g., [NOUN PHRASE1] [VERB] → [NOUN PHRASE 2] [VERB] [NOUN PHRASE1]) that allows other verbs to be used this way even if they are unattested in that form (e.g., *The stick broke* → *The man broke the stick*). A fully adult-like command of language is achieved only when such generalizations are restricted to verbs that are grammatical in the target construction; failure to do so will yield ‘over-generalization’ errors (e.g., *The woman laughed* → *The man laughed the woman). The current paper aims to elucidate the mechanisms employed by children to restrict their linguistic generalizations. Specifically, we examine young children's (age 3-4; 5-6) restrictions of verbal *un-* prefixation (e.g., squeeze→*unsqueeze); a domain that has been observed to yield overgeneralization errors in both corpus (e.g., *unbend, *uncome, *unhate, *unpress, *uncapture; [1]) and production studies (e.g., *unstick, *uncrush, *unbury, *unbend, *unsqueeze; [2]), with children as young as three years old.

The retreat from overgeneralization cannot be explained in its entirety by negative-evidence [3] which holds that these errors cease as a consequence of a caregiver's corrective feedback (e.g., if a child says *The man laughed the woman* then the caregiver may offer a correction such as *The man made the woman laugh*). Specifically, it is not feasible for every possible overgeneralization to be corrected and this position is supported by findings that overgeneralizations containing novel verbs are recognised as ungrammatical by children and adults (e.g., [4]). Rather, a number of recent findings (see [5] for review) have suggested that any theory that accounts for children's retreat from overgeneralization errors must include a role for the statistical properties of the verb itself (i.e., entrenchment; [6]), the potential competing formulations that convey the desired message (i.e., pre-emption; [7]), and the relationship between the verb's semantic properties and those associated with the construction in which it appears (e.g., [8]). However, the majority of studies supporting this view have used a grammaticality-judgment paradigm which is thought to be unsuitable for children younger than 5–6, and even children at this age showing somewhat inconsistent results ([9], [10]). Examination of whether mechanisms of pre-emption, entrenchment and verb-construction semantics are also employed by younger children is crucial to our understanding of children's retreat from overgeneralization and thus of language acquisition as a whole. Before discussing this issue, it is necessary to outline the specific factors that each of these mechanisms is assumed to involve.

In pre-emption [7], the repeated presentation of a verb in a particular construction constitutes ever-strengthening probabilistic evidence that non-attested alternative formulations which express the same intended meaning are ungrammatical. For example, transitive uses of the verb *laugh* (e.g., *The man laughed the woman) are posited to be blocked by periphrastic causative uses of that verb (e.g., *The man made the woman laugh*) because both formulations convey a similar meaning (i.e., external causation). However, the theory holds that transitive uses of *laugh* are not blocked by intransitive uses (*The woman laughed*) because the intransitive structure conveys a different meaning (internal causation). For example, children as young as 4;7 have been shown to be less likely to produce transitive sentences with novel verbs if those verbs have been modelled in the periphrastic causative construction [11]. Furthermore, evidence for pre-emption has been observed in children's (aged 5–6 and 9–10) and adults' judgments of overgeneralizations involving the dative construction (e.g., *Bart whispered Lisa the secret; [12]).

Conversely, in entrenchment [6], the repeated presentation of a verb in *any* context constitutes ever-strengthening probabilistic evidence that non-attested uses of that verb are ungrammatical. For example, transitive uses of the verb *laugh* are posited to be blocked by both periphrastic and intransitive uses of the verb (i.e., *The man made the woman laugh; The woman laughed*), and indeed any other uses (*He laughed it off*; *You’re laughing at it; Laughing!* etc.). Evidence for this theory was demonstrated by a study in which children aged 3;4 were less likely to produce transitive causative overgeneralization errors with high frequency verbs (e.g. *come*) than with low frequency verbs (e.g. *arrive*; [13]). Evidence for entrenchment has also been observed in children's (aged 5–6 and 9–10) and adults' judgments of overgeneralizations involving transitive [4]), dative [12] and locative constructions (e.g., *Marge splashed the carpet with juice; [14]).

A semantically-focused approach arises from the claim that each construction is associated with particular semantic features. For example, the transitive-causative is associated with *direct external causation* (e.g., *X broke Y*), whereas the intransitive is associated with *internal causation* (e.g., *Y broke*). Pinker's [8] semantic verb class hypothesis theorised that each verb in a speaker's lexicon is assigned to a ‘narrow-range’ semantic class, with particular classes semantically consistent with – and hence grammatical with – particular sets of constructions. For example, verbs like *ascend* and *rise* belong to a *motion in a particular direction* class that is semantically consistent with the semantics of the intransitive construction but not the transitive construction (*ascending* and *rising* can be internally caused but not directly externally caused). Conversely, verbs like *swing* and *bounce* belong to a *manner of motion* class that is semantically consistent with the semantics of both the intransitive and transitive constructions (these verbs having elements of both internal and external causation), and can thus freely alternate between the two constructions. Evidence for this proposal was demonstrated in a study which found that children as young as 4;7 were more likely to produce transitive causative sentences with novel verbs consistent with a *manner of motion class* as opposed to a *motion in a particular direction class* [11].

In its original form, Pinker's [8] discrete class-based proposal (either a verb is a member of a compatible semantic class, or it is not) does not naturally explain the finding that grammatical acceptability appears to be a probabilistic, graded phenomenon, whereby grammaticality depends on the *extent* to which a verb's semantics are consistent with those of the target construction. For example, the greater the extent to which a verb has semantic properties associated with the transitive, locative, and dative constructions, the greater the extent to which it is felicitous in those constructions, as rated by children (aged 5–6 and 9–10) and adults (e.g., [12], [14], [15]). Thus, previous literature regarding verb-argument structure overgeneralization errors points to a role for pre-emption, entrenchment and probabilistic verb-and-construction semantics.

However, the problem of retreat from overgeneralization applies not just to syntax (i.e. verb-argument structure), but to morphology as well. Additionally, a truly developmental understanding of the retreat from error can only be achieved by investigating children of all ages – including those younger than 5;0 who have been neglected by the type of judgment studies outlined above. To illustrate these points, children as young as 3;2 have been found to overgeneralize the application of *un*- prefixation to incompatible verbs (e.g.,*unbend; *uncome; [1]) and it is therefore important to examine (i) whether younger children's productivity is restricted by pre-emption, entrenchment and verb-and-construction semantics, and (ii) whether these mechanisms can be extended to the domain of morphological verbal *un*- prefixation (note that the only studies to our knowledge that have investigated the role of pre-emption, entrenchment, or verb-and-construction semantics in children less than 5 years old [11], [13], have focused on the transitive alternation).

Ambridge [16] investigated whether children's (aged 5–6; 9–10) and adults' restrictions on *un*- prefixation could be explained by the mechanisms outlined above. For pre-emption to apply to the domain of *un-* prefixation, it is necessary for ungrammatical *un-* forms (e.g., *unsqueeze) to be pre-empted by near synonyms (e.g., *release, loosen*). Thus the hypothesis predicts that errors will be less common for verbs with frequently occurring (near) synonyms to their *un-* form. In contrast, the entrenchment hypothesis holds that such errors will be less common for verbs that occur frequently without the *un-* prefix. Ambridge offered evidence that both mechanisms can be extended to the domain of verbal *un-* prefixation. Participants rated the grammaticality of 48 *un-* prefixed verb forms on a 5-point scale; half grammatical (e.g., *unbutton; unlock*), half ungrammatical (e.g., *unfill; *ungive). Frequency counts of (a) verbs in bare form (e.g., *squeez-e-es-ed-ing*) and (b) synonyms of their *un-* form (e.g., *release* and *loosen* for *unsqueeze) were obtained to examine the entrenchment and pre-emption accounts respectively. The findings for 9–10 year olds supported these hypotheses, with both frequency counts negatively predicting the rated acceptability of ungrammatical *un-* forms. However, neither entrenchment nor pre-emption were supported for the youngest children (aged 5–6). Thus, Ambridge demonstrated a successful extension of entrenchment and pre-emption to verbal *un-* prefixation, but only for later stages of development. One possibility is that sufficient entrenchment/pre-emption had not yet occurred; another is that these younger children simply struggled with the judgment task. The present study picks apart these possibilities by running a judgment task and a production task designed to be less-demanding for this age group.

How can the semantic approach be applied to verbal *un-* prefixation? Verbs that do and do not take the prefix do not appear to form discrete Pinker-style semantic classes. Rather, verbs which license *un-* cluster into a fuzzy “semantic cryptotype” of shared meanings (e.g., *covering, enclosing, attaching, circular motion, change of state, binding/locking*; [17], [18]). “Cryptotype” is a term used by Whorf to refer to a covert category of overlapping, probabilistic meanings that are difficult to access relative to overt prototypical grammatical categories (e.g., for the transitive construction). No individual feature is necessary or sufficient to license *un-* prefixation; rather, the summed expression of these features reflects each verb's compatibility with the prefix. To underline this point, Whorf noted that “we have no single word in the language that can give us a clue to its meaning;.hence the meaning is subtle, intangible, as is typical of cryptotypic meanings.”

Ambridge's [16] grammaticality judgment study of verbal *un-* prefixation examined the psychological reality of Whorf's semantic crytpotype [18]. Each of 48 test verbs were rated for the extent to which they denoted 20 semantic features hypothesised by Li and MacWhinney [17] to represent the semantic cryptotype. For all age-groups (aged 5–6, 9–10; adults), a positive correlation was observed between the extent to which a verb was compatible with the semantic cryptotype and its rated grammaticality in *un-* form, constituting clear evidence for the graded probabilistic use of verb semantics by children as young as 5–6.

To summarise, recent findings suggest a role for pre-emption, entrenchment and probabilistic verb-and-construction semantics in the retreat from overgeneralization, at least for children aged 5–6 and older. However, this research has mainly been limited to judgment studies, which themselves may be inappropriate for children younger than 5 years. Furthermore, judgment studies have yielded mixed findings for 5–6 year olds, with this age-group demonstrating effects of statistical learning (i.e., pre-emption and/or entrenchment) in judgments of transitive [4], dative [12] and locative constructions [14] but not verbal *un*- prefixation [16]. Although it is possible that children were too young for the relevant lexical items to have undergone sufficient entrenchment/pre-emption, an alternative possibility is that, for these younger children, the judgment paradigm was too demanding, insensitive or noisy to detect statistical learning effects. In the present study, we investigate the possibility that a potentially-easier experimental task - elicited production - may be more likely to detect the full range of restriction mechanisms employed by younger children. This was achieved by having the same children (aged 3–4 and 5–6) complete both a Production (Experiment 1) and Judgment study (Experiment 2).

### Ethics Statement

Experiments 1 and 2 were approved by the University of Manchester Ethics Committee. Informed written consent was obtained from the parents of the children who took part.

## Experiment 1: Production Study

### Participants

Participants were 20 children aged 3;6–4;7 (*M* = 4;0) and 20 children aged 5;6 to 6;6 (*M* = 6;0). An additional four children from the youngest age group were recruited but excluded because they did not comply with the procedure. All participants were monolingual and did not possess any known language impairment. The children were recruited from nurseries and schools in Manchester and were tested at those locations in a separate room.

### Design

Participants were divided into one of four counterbalanced groups which differed according to which verb-set was used in target sentences (*verb-set* “*A*” or “*B*”; see *Procedure and Materials*) and whether the production task (Experiment 1) preceded or followed the judgment task (Experiment 2). The dependent variable was whether or not the child produced the target verb in *un-* form on each trial. We used the same independent variables as [16] so that a fair comparison could be made with that study. The first three independent variables were employed as control measures to ensure that any effect of pre-emption, entrenchment or verb-and-construction semantics (we will henceforth use the term “semantic-cryptotype” when referring this concept in the domain of *un*- prefixation) could not be attributed to one of these measures.

- ***Corpus presence of un-form*** **(** ***Verb-type*** **).** Each test verb's existence/non-existence in *un-* form within the British National Corpus [spoken and written]; BNC) was recorded to control for the possibility that verbs which are attested in *un-* form are more likely to be produced in *un-* form. The BNC was used to obtain all frequency counts in the current study because corpora of children's speech (such as CHILDES [19]) registered many acceptable *un*- forms as having zero-frequency despite being perfectly acceptable in *un*- form.
- ***Corpus frequency of un- form (log transformed)*** **.** Each verb's frequency in *un-*form within the BNC was recorded in order to control for the likelihood that verbs that have been frequently encountered in *un-* form are more likely to be produced in this form.
- ***Reversibility Measure*** **(** ***log transformed*** **).** In order to control for the possibility that acceptability in *un*- form is simply a proxy for the reversibility of the action denoted, Ambridge [16] had 15 adult participants rate the extent to which each test verb (presented in bare form only) was reversible using a 7-point scale (see [16], for details).
- ***Pre-emption measure (log transformed)*** **.** This was the summed frequencies of the two most commonly-suggested synonyms for each verb's *un-* form (e.g., *empty* and *drain* for *unfill) in the BNC. Ambridge [16] asked 15 adults to suggest potential synonyms (other than *un-* forms) for the reversal of a verb's bare form.
- ***Entrenchment measure (log transformed)*** **.** This was simply the frequency of each verb's bare (i.e., NOT *un-* prefixed) form (all inflected forms; e.g., *fill/fills/filled/filling*) in the BNC (all texts).
- ***Semantic-cryptotype measure*** **.** This was a composite measure (created using Principal Components Analysis; PCA) of the extent to which each verb was rated (by a separate group of adults) as instantiating each of 20 semantic features proposed by Li and MacWhinney [17] to collectively characterise the semantics of verbs that may be grammatically prefixed with *un-*, based mostly on Whorf's [18] cryptotype (see [16]). The 20 semantic features were as follows (note that as a consequence of PCA, only 9 features comprised the final semantic cryptotype measure – all identified in bold font): (1) **Mental Activity**, (2) Manipulative Action, (3) **Circular Movement**, (4) **Change of location**, (5) Change of state, (6) Resultative, (7) A affects B, (8) **A touches B**, (9) A distorts B, (10) A contains B, (11) **A hinders B**, (12) A obscures B, (13) A surrounds B, (14) A tightly fits into B, (15) **A is a salient part of B**, (16) **A and B are separable**, (17) A and B are connectable, (18) **A and B are interrelated**, (19) A and B are in orderly structure, (20) **A and B form a collection**. The loadings of the nine original features on the composite semantic-cryptotype measure were as follows: Change of Location (0.92), A and B are separable (0.91), A touches B (0.78), Mental Activity (0.72), A and B are interrelated (0.71), A hinders B (0.69), Circular Movement (0.68), A is a salient part of B (0.63), A and B form a collection (−0.51).

### Procedure and Materials

The experiment used a production priming paradigm. Children were asked to take turns with the experimenter to describe cartoon picture sequences on a laptop (this arrangement allowed for the experimenter's description to serve as a ‘prime’ sentence and the child's description to serve as a ‘target’ sentence). All prime and target sentences corresponded to a cartoon sequence of an action followed by a reversal of that action. Each prime sentence was read-aloud in full by the experimenter and consisted of a verb that was grammatical in *un*- form (e.g., *Homer buckled his belt and then he unbuckled it*). The target sentence was begun by the experimenter (e.g., *Lisa squeezed the sponge and then she…*) but was completed by the child, such that she was responsible for describing the reversal action of the cartoon (e.g., …*unsqueezed/loosened/released it). Half of the target sentences contained verbs that are grammatical in *un*- form, half ungrammatical; the rationale was that children's restrictions on verbal *un-* prefixation would dictate whether the reversal action was – or was not – described with the target verb's *un-* form.

To ensure that the paradigm was age-appropriate, the experiment took the guise of a bingo game similar to that used by a recent developmental structural priming study [20] whereby a confederate would pseudo-randomly hand ‘bingo cards’ to players following a prime sentence or target sentence. The bingo cards (i.e., tokens) matched the sentence that had been spoken and served as rewards for completing a trial and thus helped keep the children engaged in the game throughout the study. The first player to fill up his or her bingo grid won the game (every session was fixed such that the participant would win the bingo game on the final target trial of the session).

#### Target Verbs

Forty-eight target sentences were created, each with a different target verb (note that to allow for the most meaningful comparison, the target verbs were the same as test verbs used in Ambridge's [16] judgment study). A check of the CHILDES database [19] – whereby we extracted frequencies at which verbs are produced by, and heard by children aged one to seven years old - revealed that the majority of the verbs used occurred frequently in child-directed speech, and – indeed – were often used by the children themselves (see Appendix S3). We thank Dave Ogden for making available to us a spreadsheet containing the frequencies of each individual lexical item in the entire CHILDES database. It is also worth noting that in even our Judgment study (which is a relatively difficult task for young children), examination of “zero” verbs (i.e., those that cannot take *un*-) revealed that each age-group misidentified no more than three of these verbs as being more acceptable in *un*- form than their bare form – see *Figure S1* and *Figure S2*. Additionally, all verbs were accompanied by picture sequences to demonstrate each verb's meaning (in both Experiment 1 and 2), and on no occasions did children indicate to the researcher that they were unsure of a verb's meaning. Thus, we can be confident that most children were familiar with and understood the majority of these verbs (allowing us to use the same set as Ambridge [16] - so as to ensure comparability across studies).

Half of the target verbs were grammatical in *un-* form (*“un-* verbs”) and half ungrammatical in *un-* form (“*zero-* verbs”), as classified by Li and MacWhinney [17]:

- ***un- verbs (N = 24)***: Bandage, Buckle, Button, Chain, Cork, Crumple, Delete, Do, Fasten, Hook, Lace, Latch, Leash, Lock, Mask, Pack, Reel, Roll, Screw, Snap, Tie, Veil, Wrap, Zip.
- ***zero- verbs (N = 24)***: Allow, Ask, Believe, Bend, Close, Come, Embarrass, Fill, Freeze, Give, Go, Lift, Loosen, Open, Press, Pull, Put, Release, Remove, Sit, Squeeze, Stand, Straighten, Tighten.

It is important to note that nothing hinges on the accuracy of this classification of verbs as *un-/zero* (the classification was not used as a predictor in any statistical analysis). The point is simply that roughly half of the target *un-* forms were broadly-speaking grammatical, meaning that children could not usefully adopt a task-dependent strategy of treating all as grammatical (or ungrammatical). In order to reduce the number of trials completed by children, each child was assigned only one of two sets of 24 target verbs (Verb-set A/Verb-set B; see Appendix S1), each containing 12 randomly selected *un-* verbs and 12 randomly selected *zero* verbs.

#### Prime Verbs

There were also 24 prime sentences for each participant with the caveat that no verb served as both a prime and target verb for the same participant. Thus, the 12 grammatical *un-* verbs used as target verbs in Verb-set A were used as prime verbs for Verb-set B, and vice versa. Twelve additional verbs (mostly taken from Li and MacWhinney [17] and all grammatical in *un-* form) were used as prime verbs for all participants, in order to make up the total of 24 primes per participant.

#### Sentences

For each verb (both prime and target) we created a sentence of the form *[CHARACTER] [VERB-ed] and then (s)he un-[VERB-ed]* (see Appendix S1 for a full list), and a corresponding sequence of still cartoon pictures. Four different characters (*Homer, Bart, Lisa* and *Marge*) were used. An additional three prime and target sentences plus corresponding sequences were created for the practice session; all used verbs that were grammatical in *un-* form (this served to encourage production of *un*- forms before testing began) and did not form part of the test sets. The prime and target sentences were randomly selected for each trial; we did not use pre-specified prime and target pairs. To avoid the task becoming too arduous for children, the test session was divided into two sessions of 12 prime-target trials, with a rest period between each session.

### Coding

Coding was based on the child's first response only. Responses were coded as “*un-* form”, “not *un-*” or “other” (i.e., excluded) according to the following criteria:

- ***“Un-form”:*** if the target verb was produced with *un-* prefixation (e.g., EXP: *Homer wrapped the present and then he…* CHI: *unwrapped it*).
- ***“Not un-”:*** if the participant described the reversal action accurately without using the target verb in *un-* form (e.g., *took the wrapper off*).
- ***“Other”*** *:* Responses were excluded from analyses if: (i) there was experimenter error, or (ii) the response did not accurately describe a reversal of the action denoted by the target verb; this criteria includes responses in which a general reversal term (e.g., *didn’t*) was used without any relevance to the specific reversal action (e.g., *Marge allowed Bart some chocolate and then she…didn’t*).

### Results and Discussion

The current study used an elicited production paradigm to investigate children's (aged 3–4; 5–6) grammatical restrictions on verbal *un-* prefixation. Collapsing across all verbs, responses were coded as “Other” for 9.79% of 3–4 year olds' trials and 4.38% of 5–6 year olds' trials (out of 47 trials excluded for 3–4 year olds, 35 were due to the child's response being an inaccurate description of the reversal action, or use of a general reversal term [e.g., “*didn’t*”], 10 were due to no response being given, and 2 were due to experimenter error; out of 21 trials excluded for 5–6 year olds, 10 were due to an inaccurate description of the reversal action or use of a general reversal term, 5 were due to no response being given, and 6 were due to experimenter error). Once these trials were excluded from the denominator, 3–4 year olds and 5–6 year olds produced *un-* forms of the target verb on 37.64% (SD =  48.5) and 69.06% (SD = 46.27) of trials respectively. Given (a) the low rate of excluded “Other” responses, and (b) the fact that only around 50% of target verbs are grammatical in *un-* form, these totals indicate that the production priming paradigm was highly successful at eliciting both *un-* forms and alternative reversal verbs. Furthermore, examination of *zero*-verbs only (i.e. verbs that do not take *un*-) revealed that the younger age group produced *un*- forms on 23.38% [SD = 42.46] of these trials (older group = 50.31% [SD = 50.16]). Thus, we can also be sure that both age-groups were over-generalizing *un*- prefixation to verbs that do not take un (i.e., *zero*-verbs).

Results were analysed using binomial linear mixed effects models (*lmer* from package *lme4*; [21]) in the *R* environment [22]). Mixed-effects models predict individual trials rather than averaging over trials, and offer the added benefit of treating both participant and item as random effects (i.e., the model creates an intercept for each participant and each item, thus removing variation within each of these factors). They are also robust against missing data [23]). The outcome variable was whether the child produced a “*un-*” or “not*-un*” response on each trial (“other” responses were excluded). Fixed effects were measures of (a) *verb-type* (b) *frequency of verb in un- form*, (c) *reversibility*, (d) *pre-emption*, (e) *entrenchment*, and (f) *semantic-cryptotype* (see *Method* section for details). All models included random intercepts for participants and verbs. Adding random slopes made no significant difference (p>0.05) to any model's coverage of the data. Although some researchers have argued that random slopes should be included in all cases (e.g., [24]), this conclusion is by no means accepted by all experts in mixed effects modelling (e.g., [25]); thus, the models reported below do not include random slopes. In line with the recommendations of a recent paper [26], we used simultaneous regression models with neither residualization nor centering. The models for each age-group are shown in *Table 1* (because all predictors were entered in a single step, the order in which they are listed is arbitrary). A positive beta (*β*) value indicates a positive correlation between the predictor and the likelihood of a verb being produced in *un-* form – as expected for *semantic-cryptotype*. A negative *β* value indicates a negative correlation between the predictor and the likelihood of a verb being produced with *un-* prefixation – as expected for measures of *pre-emption* and *entrenchment*.

**Table 1:** Mixed Effects Models for production data (Age 3*-*4; Age 5*-*6).

				Age 3–4						Age 5–6		
Fixed effects	*Beta (â)*	*SE*	*z value*	*HPD95 lower*	*HPD95 upper*	*p*	*Beta (â)*	*SE*	*z value*	*HPD95 lower*	*HPD95 upper*	*p*
(Intercept)	4.29	1.90	2.26	0.57	8.01	0.02	6.81	1.84	3.70	3.20	10.42	0.00
Verb Type	−0.81	0.68	−1.19	−2.14	0.52	0.23	−0.78	0.67	−1.18	−2.09	0.53	0.24
Freq of *un*- form	**0.25**	**0.12**	**2.05**	**0.01**	**0.49**	**0.04**	**0.42**	**0.13**	**3.13**	**0.17**	**0.67**	**0.00**
Reversibility	−0.35	0.32	−1.09	−0.98	0.28	0.28	−**0.87**	**0.32**	−**2.70**	−**1.50**	−**0.24**	**0.01**
PreEmption	−**0.16**	**0.08**	−**2.01**	−**0.32**	**0.00**	**0.04**	**-0.19**	**0.08**	−**2.53**	−**0.35**	−**0.03**	**0.01**
Entrenchment	−**0.33**	**0.12**	−**2.76**	**-0.57**	−**0.09**	**0.01**	−0.21	0.13	−1.57	−0.46	0.04	0.12
Semantics	0.34	0.25	1.35	−0.15	0.83	0.18	0.37	0.28	1.34	−0.18	0.92	0.18

#### Age 3–4

**Figure 1:**
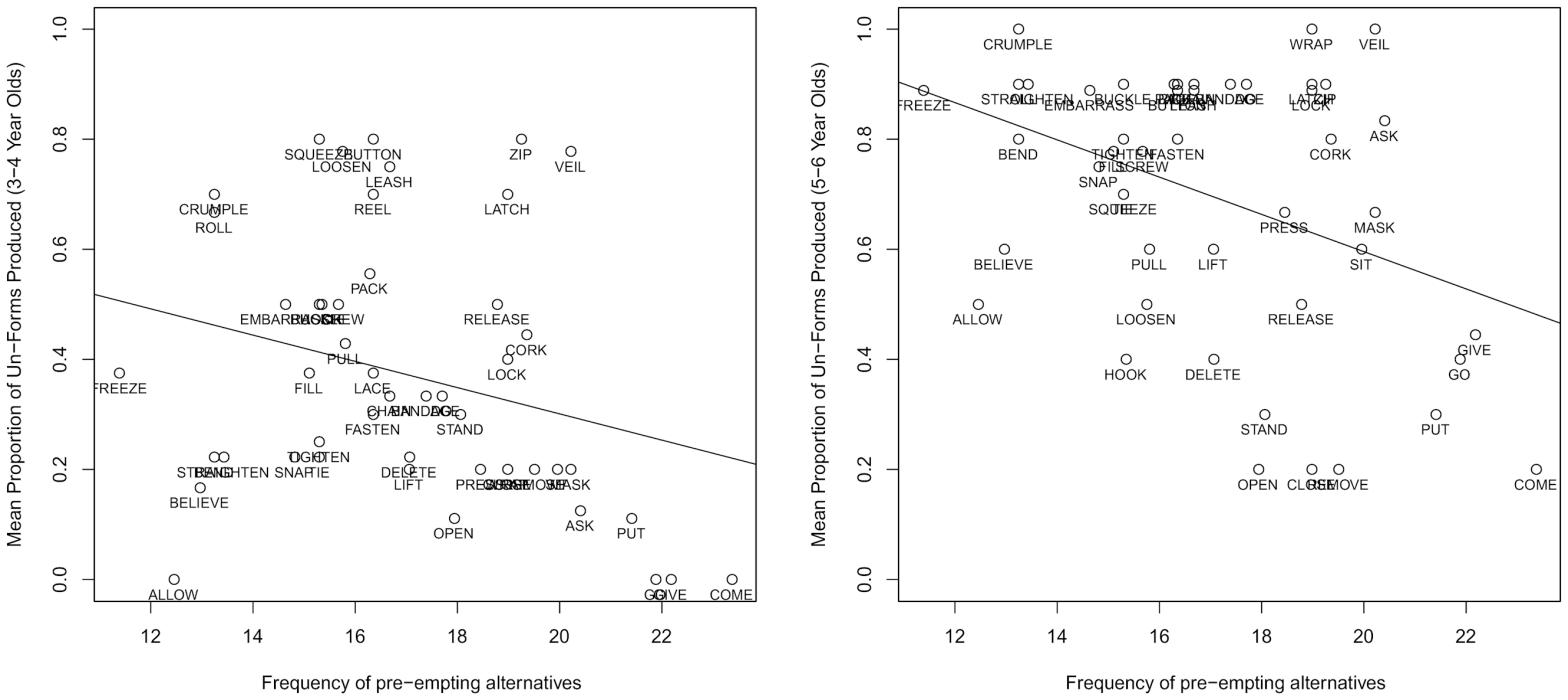
Mean proportion of *un-* forms produced for each verb by age group as a function of the pre-emption predictor (age 3–4 on the left; age 5–6 on the right).

Considering first the control predictors, a main effect of *frequency of un- form* was observed, such that production of *un*- forms was positively related to the target verb's corpus frequency in *un*- form. The other control predictor – *reversibility* – did not exert any significant effect, indicating that the study's *semantic-cryptotype* measure did not serve as a proxy for reversibility. Turning now to the predictors of interest, production probability of *un*- forms was negatively related to the frequency of both pre-empting forms (*pre-emption*; see Figure 1), and the verb's bare form (*entrenchment*).

#### Age 5–6

Again considering first the control predictors, a main effect of *frequency of un- form* was observed, such that production of *un*- forms was positively related to the target verb's corpus frequency in *un*- form. Interestingly, a *negative* effect of *reversibility* was observed, such that *un*- forms were more likely to be produced with verbs that were *less* reversible – this emphasises that the *semantic*-*cryptotype* measure could not have been a proxy for a verb's reversibility. Turning now to the predictors of interest, a significant negative correlation was observed between the proportion of *un*- forms produced and *frequency of pre-empting forms* (*pre-emption*; see Figure 1), but not entrenchment.

The results outlined above demonstrate that both 3–4 year olds and 5–6 year olds use *pre-emption,* such that production of *un-* forms was negatively predicted by corpus frequency of synonyms for the target verb's *un-* form. An effect of *entrenchment* – such that production of *un-* forms was less likely when the target verb was highly frequent in bare form - was observed for 3–4 year olds but not 5–6 year olds. The effect of *semantic-cryptotype* failed to reach significance for either age-group.

The finding of no *semantic-cryptotype* effect for the 5–6 year olds is at odds with that of Ambridge [16] who did find such an effect. A possible explanation for this pattern is that – for older children - a grammaticality judgment task – as used in this previous study – is better suited to detecting fine-grained semantic effects than is a binary production task. On the other hand, an effect of *pre-emption* for 5–6 year olds was observed in the present study, but not the judgment study of Ambridge, possibly because the *semantic-cryptotype* effect observed in this previous study left less variance to be explained by *pre-emption*. Another possibility is that a production task encourages children to search their lexicon for pre-empting alternatives to ungrammatical *un-* forms to a greater extent than does a judgment task.

In order to examine these possibilities, and to investigate the relationship between production and grammaticality judgment data more generally, we investigated whether the children who participated in the current production study would show a similar pattern of data in a grammaticality judgment task.

## Experiment 2: Grammaticality Judgment Study

### Participants

Participants were the same as those who took part in the production study. The two studies were completed at least one week apart, in counterbalanced order.

### Design

Participants remained in their counterbalanced groups (e.g., participants exposed to Verb-set A in the production study were asked to judge the grammaticality of target verbs from that set, in both *un-* and bare form). The dependent variable was the acceptability rating of each *un-* form on a scale of 1 to 5 (log transformed). The judgment study used the same predictor variables as the production study, plus one additional predictor:

- ***Acceptability of bare form (log transformed).*** Participants rated the acceptability of each verb's bare form (e.g., *squeeze*) to control for the possibility that individual participants would show general (dis)preferences for particular verbs, perhaps based on semantic or phonological properties, regardless of form (*un-*/bare).

### Procedure and Materials

**Figure 2:**
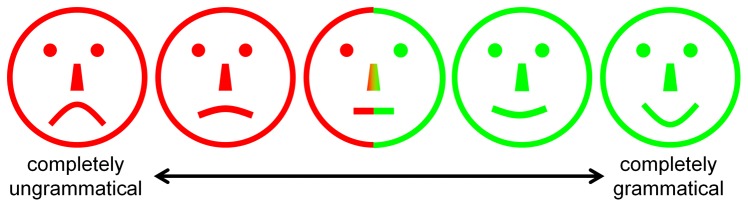
The 5-point smiley face scale used by participants to rate the relative acceptability of the *un-* prefixed and bare verb forms (reproduced from Ambridge et al., 2008: 105, by permission of Elsevier).

All sentences were presented in audio form. To make the task more engaging, children were introduced to a toy dog that was ‘learning to speak English.’ The child was asked to help the dog to speak properly by telling him which words sounded “right” and which words sounded “wrong and a bit silly” (for full details see, [4], [16]). Children were then introduced to a five-point smiley-face scale (*Figure 2*) which would be used to rate sentences in a graded manner. In short, the process involved a child taking a green or red counter to indicate grammatical or ungrammatical items respectively and placing the counter on the scale to indicate the degree of grammaticality (5 = perfectly grammatical; 1 =  very ungrammatical). To familiarise themselves with the rating scale, participants first completed a practice session comprised of seven sentences, each including either a correct past-tense forms or an over-regularization error (e.g., *Homer breakded the cup), accompanied by an appropriate picture sequence. Participants were asked to rate the *verb only*: After the participant had heard the full sentence, the experimenter repeated the verb in isolation and asked participants to indicate its grammaticality). The subsequent two test sessions took the same format as the practice session.

### Sentence Stimuli

Each verb was presented in two separate trials: once in bare form to obtain a control rating (e.g., *Lisa squeezed the sponge*) and once in *un-* form (*Lisa unsqueezed the sponge). There were thus 96 test trials (48 bare- and 48 *un-* forms) in the judgment study as opposed to just 48 in the production study. Children remained in their counterbalanced groups (Verb-set A or Verb-set B) and were thus only required to complete 48 judgment trials each (24 bare forms and 24 *un-* forms). The test session was split into two separate sessions of 24 trials to avoid the task becoming too arduous. Each test session contained a verb's bare and *un-* form but these forms were never presented in consecutive trials; with this caveat in mind, all trials were presented in a random order for each participant. For a full list of practice and test sentences, see Appendix S2.

### Results and Discussion

The purpose of the grammaticality judgment study was to examine the possibility that, compared with a production task, a judgment task is more likely to detect fine-grained semantic-cryptotype effects (due to its greater sensitivity). As well as an exploration of the relationship between production and judgment data more generally, it also served as an investigation of whether the graded grammaticality judgment paradigm could be extended to children aged 3–4.

Results were again analysed using linear mixed effects models. The dependent variable was the acceptability rating for each verb's *un-* form. All models included random intercepts for participants and verbs. The models used the same fixed effects as the production study, plus one additional fixed effect which was employed as a control variable: *acceptability ratings for each verb*'*s bare form*. Results of the judgment analyses by age group are shown in *Table 2* *.*

**Table 2:** Mixed Effects Models for Judgment Data (Age 3–4; Age 5–6).

				Age 3–4						Age 5–6		
	*Beta (â)*	*SE*	*t*	*HPD95 lower*	*HPD95 upper*	*p*	*Beta (â)*	*SE*	*t*	*HPD95 lower*	*HPD95 upper*	*p*
(Intercept)	0.82	0.49	1.65	−0.29	2.04	0.10	1.95	0.50	3.91	0.96	2.92	0.00
Verb Type	−0.13	0.20	−0.65	−0.67	0.33	0.52	−0.10	0.19	−−0.51	−0.48	0.26	0.61
Freq of *un*- form	**0.09**	**0.04**	**2.39**	**0.01**	**0.18**	**0.02**	**0.11**	**0.04**	**3.08**	**0.04**	**0.18**	**0.00**
Rating For Bare Form	**0.22**	**0.05**	**4.77**	**0.10**	**0.31**	**0.00**	**0.15**	**0.05**	**3.01**	**0.04**	**0.24**	**0.00**
Reversibility	−0.10	0.09	−1.03	−0.30	0.13	0.30	−0.09	0.09	−0.94	−0.25	0.09	0.35
PreEmption	−0.03	0.02	−1.54	−0.09	0.01	0.13	−**0.09**	**0.02**	−**4.19**	−**0.13**	−**0.04**	**0.00**
Entrenchment	0.00	0.04	−0.02	−0.08	0.09	0.99	0.02	0.04	0.68	−0.05	0.09	0.50
Semantics	0.11	0.07	1.44	−0.06	0.28	0.15	**0.32**	**0.07**	**4.26**	**0.16**	**0.45**	**0.00**

#### Age 3–4

Judgment data for the youngest group revealed no significant effects of *semantic-cryptotype*, *pre-emption* or *entrenchment*. Rather, the only significant predictors of grammaticality ratings for *un-*forms were *frequency of un- form*, and *ratings for bare form*. The former finding suggests that, while they do not yet show effects of *entrenchment* or *pre-emption* in a judgment task, 3–4-year-olds' judgments are still sensitive to at least some surface statistical properties of the input (i.e., the frequency of particular attested forms). In general, however, it seems that the judgment paradigm underestimated 3–4 year olds' grammatical knowledge (relative to the production study), given that it failed to yield any significant effects of *pre-emption, entrenchment* and *semantic-cryptotype,* the former two of which were present for the same participants in the production study. Thus, it is likely that judgment data from the younger age group were too noisy for detection of any mechanisms of restriction.

#### Age 5–6

**Figure 3:**
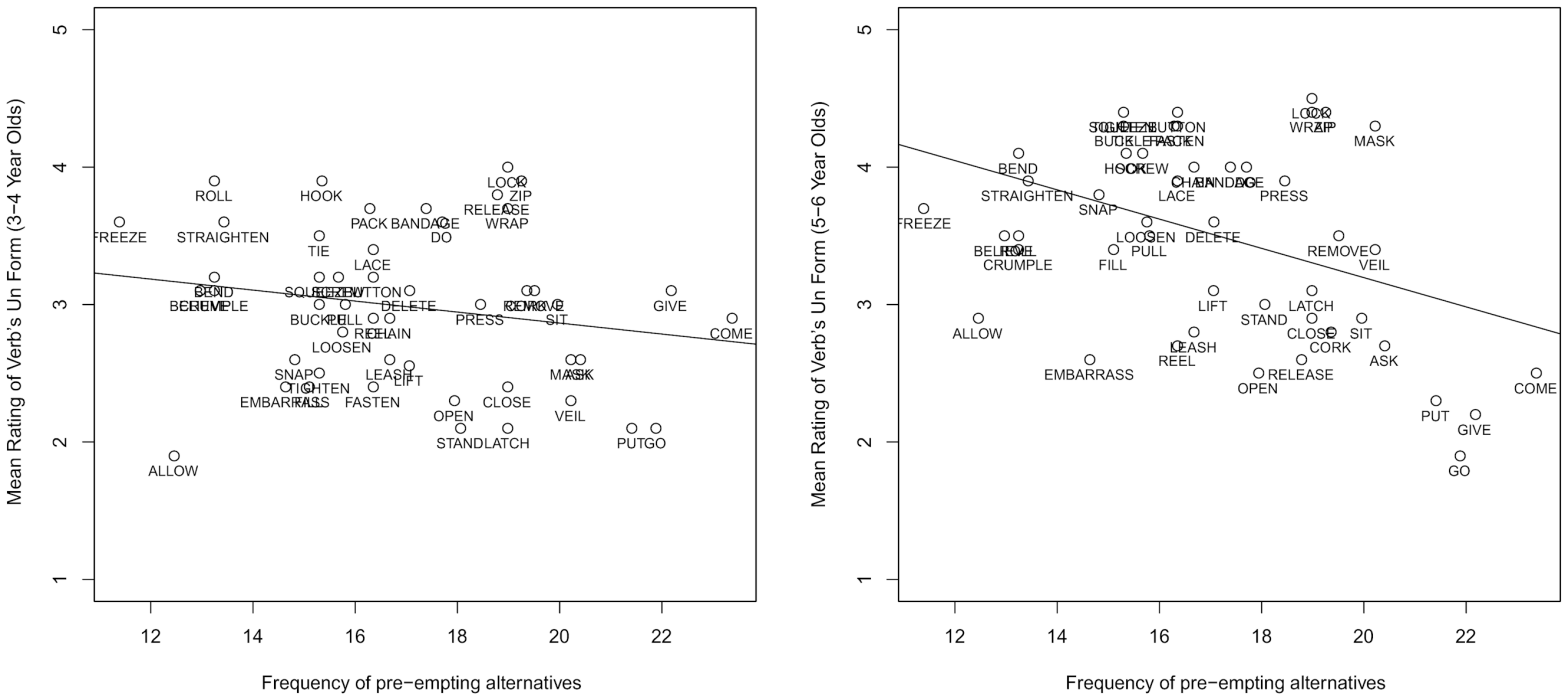
Mean acceptability rating for each verb's *un*- form as a function of the pre-emption predictor (age 3–4 on the left; age 5–6 on the right).

Considering first the control predictors, the older age group's judgments of *un-* forms were positively correlated with *frequency of un- form*, and *rating for bare form*. Turning to the predictors of interest, judgments of a verb's *un-* form were positively correlated with the extent to which verbs denoted semantics of *un-* prefixation (*semantic-cryptotype*) and negatively correlated with the frequency of pre-empting forms (*pre-emption;* see *Figure 3*). There was no effect of the *entrenchment* measure.

In summary, 3–4 year olds' judgment data appeared too noisy to yield any effects any of the proposed restriction mechanisms. Thus our knowledge of this age-group's restriction mechanisms must be taken from production data, which revealed effects of *entrenchment* and *pre-emption*, but not *semantic-cryptotype*. The older age-group (5–6 year olds) used both *pre-emption* and *semantic-cryptotype* to guide grammaticality judgments of *un-* prefixed verbs. The *pre-emption* effect persisted in this age-group's production data but the *semantic-cryptotype* effect did not, possibly because semantic effects are more fine-grained and thus harder to detect in production tasks. Taken together, Experiment 1 and 2 indicate that children as young as 3–4 are using *pre-emption* and *entrenchment* to guide productivity of verbal *un*- prefixation, and that use of a *semantic cryptotype* – a category that encompasses the semantics shared by verbs that have previously appeared in the same context – has emerged by 5–6 years old.

### Comparison between Judgment and Production Data

We suggested above that judgment paradigms may be an unsuitable measure of 3–4 year olds' grammatical knowledge. To examine the validity of this claim, we compared judgment data and production data. We expected to find a correlation between production probability and judgments of *un-* forms for 5–6 year olds, but not 3–4 year olds, on the assumption that only for the older group is the judgment paradigm a suitable measure of the grammatical knowledge that drives production

For both age groups, we ran a mixed-effects model with children's mean proportion of *un-* forms produced (i.e. production data) as the outcome measure and ratings of a verb's *bare form* (a control variable for judgment data) and *un-* form (the predictor of interest) as fixed effects. All models included participants and items as random effects.

#### Age 3–4

Younger children's production of *un-* forms was negatively predicted by their *ratings for bare forms*, *β*  = −0.45 (*SE* = 0.15), *p* = 0.003, but was not predicted by their *ratings for un- forms*, *B* = −0.00, *SE* = 0.14, p = 0.97. These data suggest that 3–4 year old children's ratings of *un-* prefixed verbs were determined by baseline (dis)preference for individual verbs (in their canonical bare form) rather than their knowledge of restrictions on *un-* prefixation, rendering the grammaticality judgment paradigm unsuitable for younger children (at least, for this particular study). Recall that the production data did indeed suggest knowledge of restrictions on *un-* prefixation for this age group.

#### Age 5–6

Older children's production of *un-* forms was not related to their *ratings for bare form*, *β* = −0.24 (*SE* = 0.16), *p* =  0.15) but was positively predicted by *ratings for un- form*, *β* = 0.34 (*SE* = 0.15), *p* = 0.023, such that the more likely a verb was rated as grammatical in *un-* form, the more likely they were to produce that verb in *un-* form.

We can conclude that that the judgment paradigm was unsuitable as a measure of 3−4 year old children's grammatical knowledge. The judgment paradigm can be considered a reasonably valid measurement of 5−6 year olds' grammatical knowledge given that judgments of verb *un-* forms predicted the likelihood a verb would be produced in *un-* form. Moreover, the paradigm yielded effects of *pre-emption* and *semantic-cryptotype* for this age-group, the latter of which was not detected by the production paradigm. Thus, when used with a suitable age-group, the judgment paradigm may be a more sensitive measure of children's use of a semantic-cryptotype in their restriction of *un*- prefixation.

## General Discussion

Recent research has demonstrated that any complete account of the retreat from overgeneralization must incorporate roles for pre-emption, entrenchment and verb-and-construction semantics (e.g., [12], [14], [15], [16]). However, the roles played by these mechanisms in the early stages of retreat from error are less clear. In the Introduction, we outlined a recent grammaticality judgment study of overgeneralization errors involving verbal *un*- prefixation [16], in which 5–6 year old children demonstrated use of a semantic “cryptotype” hypothesised to represent verbs that take *un-* [18], but no use of pre-emption or entrenchment. The current study investigated the possibility that judgment paradigms may underestimate young children's grammatical knowledge, and hence obscure pre-emption/entrenchment effects that may be present at this age and younger. To address this possibility, we employed what we hope was a less demanding production paradigm to examine young children's (3–4; 5–6) restrictions on verbal *un-* prefixation.

In Experiment 1, children were asked to describe reversal actions of verbs that were or were not grammatical in *un*- form (e.g., unwrap; *unsqueeze), the rationale being that the likelihood of that verb being produced in *un-* form would be dictated by the verb's semantic properties, its entrenchment in other contexts, and/or the frequency of pre-empting formulations. In Experiment 2, the same children were asked to give grammaticality judgment ratings for each verb's *un-* form so that findings from production and judgment paradigms could be compared.

Looking first at 3–4 year old children, production of *un-* prefixed verbs was negatively predicted by (a) the frequency of synonyms to the target verb's *un*- form (e.g., *release + loosen* for *unsqueeze) and (b) the target verb's frequency in bare form (i.e. not *un*- form; e.g. *squeez/e/s/d/ing*) – demonstrating use of pre-emption and entrenchment respectively. Thus, production data provides clear evidence that pre-emption and entrenchment are indeed operational for children as young as 3–4 (M = 4;0). However, 3–4 year olds' judgment data were deemed too noisy to detect any use of restriction mechanisms.

Examination of 5–6 year old children's data revealed that the pre-emption measure predicted judgments and production of *un-* prefixed verbs, confirming that use of this mechanism persists into this later developmental stage. A semantic-cryptotype effect was evident amongst 5–6 year olds, such that judgments of *un*- prefixed verbs were positively related to the extent to which each verb denoted a semantic cryptotype hypothesised to represent properties shared by verbs that licence *un-* (e.g. [18]).

Taken together, the present experiments indicate a role for pre-emption, entrenchment and verb-and-construction semantics from an early age. Further, it appears that children may initially learn verbs' restrictions by monitoring the distributional patterns of the verb in other contexts [entrenchment], as well as those of the verb's competing formulations that convey similar meaning [pre-emption], with a role for verb-and-construction semantics (or more specifically, in this study's case, Whorf's [18] hypothesised “semantic cryptotype”) emerging by 5–6 years old. Although it may be tempting to conclude that these results support a “statistics-before-semantics” approach whereby use of a verb's statistical properties precedes use of its semantic properties (e.g., [27]), caution must be taken in adopting this perspective. The reason is that both pre-emption and entrenchment have underlying semantic motivation. For pre-emption to operate, a speaker must recognise that a pre-empting alternative exhibits appropriate semantics that convey the same message as the target verb's *un*- form. Entrenchment can also be argued to have underlying semantic motivations, since any lexical item's entrenchment is a consequence of a verb exhibiting suitable semantics to convey the desired message (when placed in a suitable sentence construction). Thus, evidence for children's use of entrenchment or (especially) pre-emption demonstrates the ability to use a verb's statistical and – in some sense - semantic properties to restrict productivity, with the current study indicating that this ability is evident from 3–4 years old. Acknowledging previous literature that demonstrates pre-emption, entrenchment and verb-and-construction semantics to persist into later stages of development (e.g., [14], [15], [16]), it is clear that children's restriction mechanisms involve an interactive process in which ‘statistical’ and ‘semantic’ effects cannot be picked apart so easily.

One framework that may be useful for understanding these results is the *FIT* account outlined in Ambridge and Lieven [28]. A more detailed description of how this account can yield entrenchment, pre-emption and verb-and-construction semantic effects in the domain of *un*- prefixation is given in Ambridge [16]. In brief, the central idea is that all constructions in a speaker's lexicon compete for activation [29]; i.e., for selection to express the speaker's intended message (e.g., the reversal of a squeezing action). The most relevant “constructions” in this context are whole words (e.g., *release, loosen*) and the morphological *un*- prefixation construction (*un-[VERB]*).

The account yields pre-emption effects because the greater the frequency of competing forms (e.g., *release, loosen*), the greater their activation, and hence the lower the activation of the competing potential *un-* form (e.g., *unsqueeze). The account yields entrenchment effects due to the assumption that *every* construction in the speaker's inventory competes for selection, with the activation determined by – amongst other things – their relevance to the speaker's message. For example, if the message is the reversal of a squeezing action, the competitors will be not only *release, loosen* and *unsqueeze*, but *squeeze* itself. Entrenchment effects occur because the activation of each alternative is determined not only by its relevance, but also its input frequency (and hence the strength of its trace in the lexicon). Because pre-empting forms (e.g., *release, loosen*) are better (i.e., more relevant) competitors for a given *un*- error (e.g., *unsqueeze) than are entrenching forms (e.g., *squeeze*), this account may be able to explain the present finding that pre-emption appears to be more important than entrenchment. Future modelling work should attempt to clarify whether or not such an account can in fact yield this pattern (for preliminary modelling work in this domain, see [17], [30]).

The account yields verb-and-construction semantic effects due to the assumption that the *un-[VERB]*construction, like all abstract constructions, is acquired by abstracting across memory-traces of stored exemplars of this construction in memory (e.g., [31]), in this case, individual *un*-forms (*unscrew, unbutton* etc.). Thus the *[VERB]* slot of this construction probabilistically exhibits the averaged semantic properties of every item that has previously occupied that slot (e.g.,[32]). The greater the overlap between the semantic properties of this slot and a putative filler (e.g., *squeeze*), the greater the activation of the relevant *un*- form. Again, preliminary computational models of the acquisition of *un-* prefixation ([16], [31]), show this type of semantic generalization. We are agnostic with regard to the question of whether the *un-[VERB]* construction is represented independently of the exemplars that instantiate it (i.e., between prototype and exemplar models). However, the assumption that a prerequisite for this generalization is a set of stored exemplars, may be able to explain the present finding that statistical effects appear to emerge before verb-and-construction semantic effects (though – as we have just seen – not before *all* types of semantic effect): Effects of pre-emption and entrenchment can arise on the basis of the stored exemplars themselves; verb-and-construction semantic effects only as the result of some kind of generalization or analogy across these exemplars. However, to address this question more definitively, more modelling work will be needed.

One issue that we should address is that the lack of filler trials in the production study (such that all prime sentences featured reversal actions described with a *un*- prefixed verb) may have led to an unrealistic ‘over’-production of *un*- forms that was not representative of levels of *un-* prefixation in children's spontaneous speech. However, this paradigm was indeed designed to pull children towards using *un*- prefixation, the rationale being that a child's command of verbs' distributional and semantic properties should guide their productivity, thus providing a window into restriction mechanisms employed by these children. Since we obtained a number of results that differentiated between verbs, the use of this method appears to be justified. Indeed, using a method that led to lower rates of *un-* prefixation would most likely have significantly reduced the possibility of observing the by-verb differences that are required in order to test the pre-emption, entrenchment and verb-and-construction semantics hypotheses. We must also acknowledge that – on the one hand – only a judgment paradigm was sufficiently sensitive to detect semantic effects in 5–6 year olds, but – on the other – only a production paradigm was sufficiently simple to detect pre-emption and entrenchment effects in 3–4 year olds. Thus a profitable direction for future research is to employ paradigms such as eye-tracking or Event Related Potentials (ERP), that are sufficiently sensitive to detect fine-grained effects, but that can be combined with tasks that are very simple for young children.

In conclusion, the present findings indicate that children as young as 3–4 are guided by pre-emption and entrenchment in their production of verbal *un*- prefixation. By age 5–6, children also show use of a complex ‘cryptotype’ of semantic properties thought to be representative of verbs that licence *un*-. Together, these findings reflect a complex interaction between statistical and semantic properties of competing lexical items that we have posited to be operational within one interactive framework.

## Supporting Information

Figure S1 **Mean Difference Scores for 3–4 Year Olds.** Mean difference scores were calculated by subtracting the mean rating for each verb's *un*- form from the mean rating for each verb's bare form. If mean difference scores for verbs that do not take *un*- (i.e. “zero” verbs – defined by whether or not they had appeared in *un –*form in BNC) fell below the value of zero then we assert that the child did not understand the meaning of the verb; using this rationale, 3–4 year old children rated only three “zero” verbs as more grammatical than their bare form equivalent (*release, remove, straighten*) and thus we can be confident that test verbs used in the current study were suitable for use with these children.
(TIF)

Figure S2 **Mean Difference Scores for 5–6 Year Olds.** Mean difference scores were calculated by subtracting the mean rating for each verb's *un*- form from the mean rating for each verb's bare form. If mean difference scores for verbs that do not take *un*- (i.e. “zero” verbs) fell below the value of zero then we assert that the child did not understand the meaning of the verb. Five-to-six year old children rated one “zero” verbs as more grammatical than its bare form equivalent (*squeeze*). Thus, we can be confident that test verbs used in the current study were suitable for use with this age-group.
(TIF)

Data S1 **Data used in this study.** Data are available in the supporting file titled Data S1.
(XLSX)

Appendix S1 **Practice and Test Sentences for Production Study.**
(DOCX)

Appendix S2 **Practice and Test Sentences for Judgment Study.**
(DOCX)

Appendix S3 **CHILDES Frequency Counts of Each Verb**
(DOCX)
