# Supplementary figures and images for: Children Use Statistics and Semantics in the Retreat from Overgeneralization
Source: PLoS One. 2014 Oct 15;9(10):e110009. doi: 10.1371/journal.pone.0110009 (PMC4198212; doi:10.1371/journal.pone.0110009)

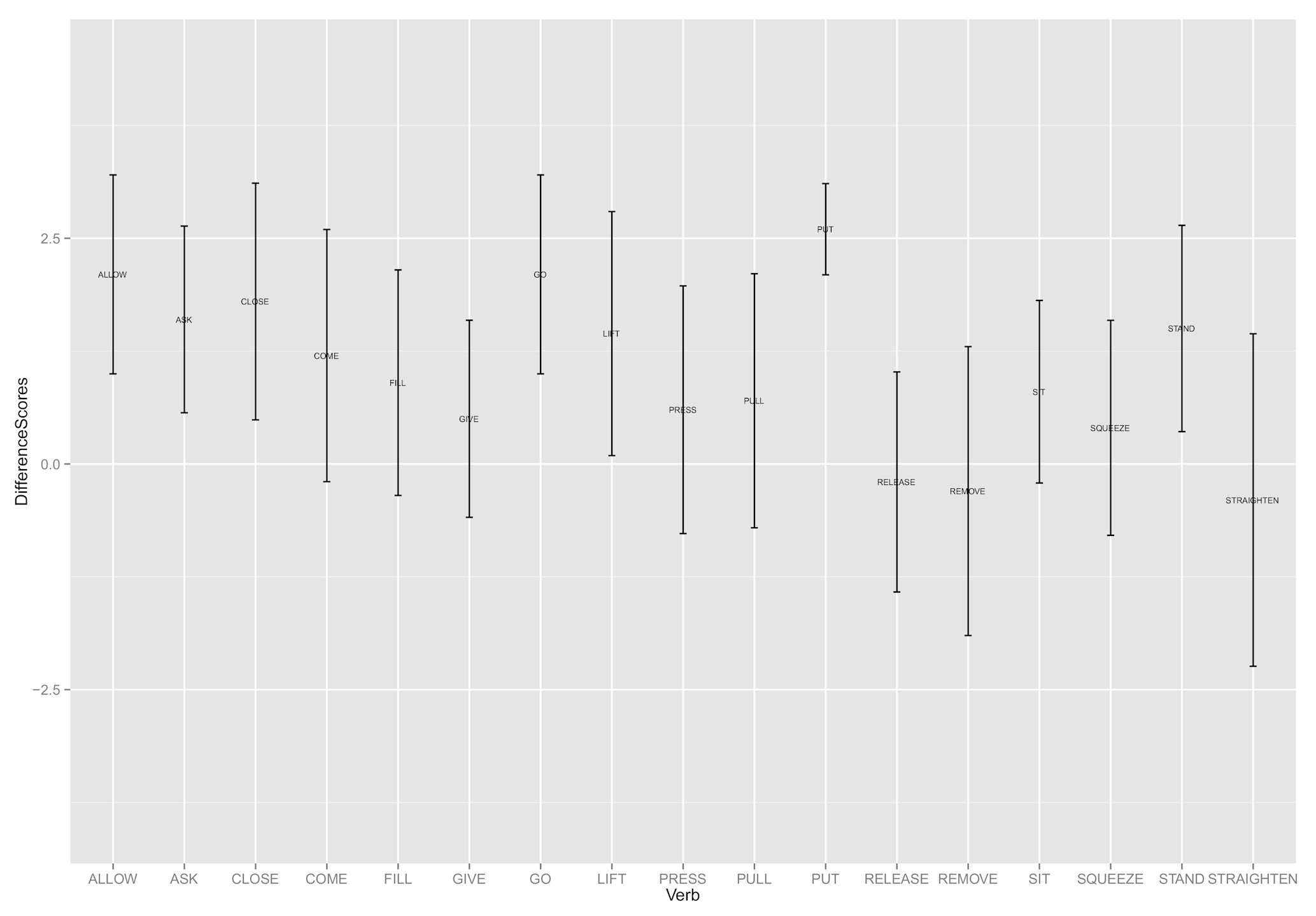

Supplement: Figure S1 — Mean Difference Scores for 3–4 Year Olds. Mean difference scores were calculated by subtracting the mean rating for each verb's un- form from the mean rating for each verb's bare form. If mean difference scores for verbs that do not take un- (i.e. “zero” verbs – defined by whether or not they had appeared in un –form in BNC) fell below the value of zero then we assert that the child did not understand the meaning of the verb; using this rationale, 3–4 year old children rated only three “zero” verbs as more grammatical than their bare form equivalent (release, remove, straighten) and thus we can be confident that test verbs used in the current study were suitable for use with these children. (TIF) [file pone.0110009.s001.tif]

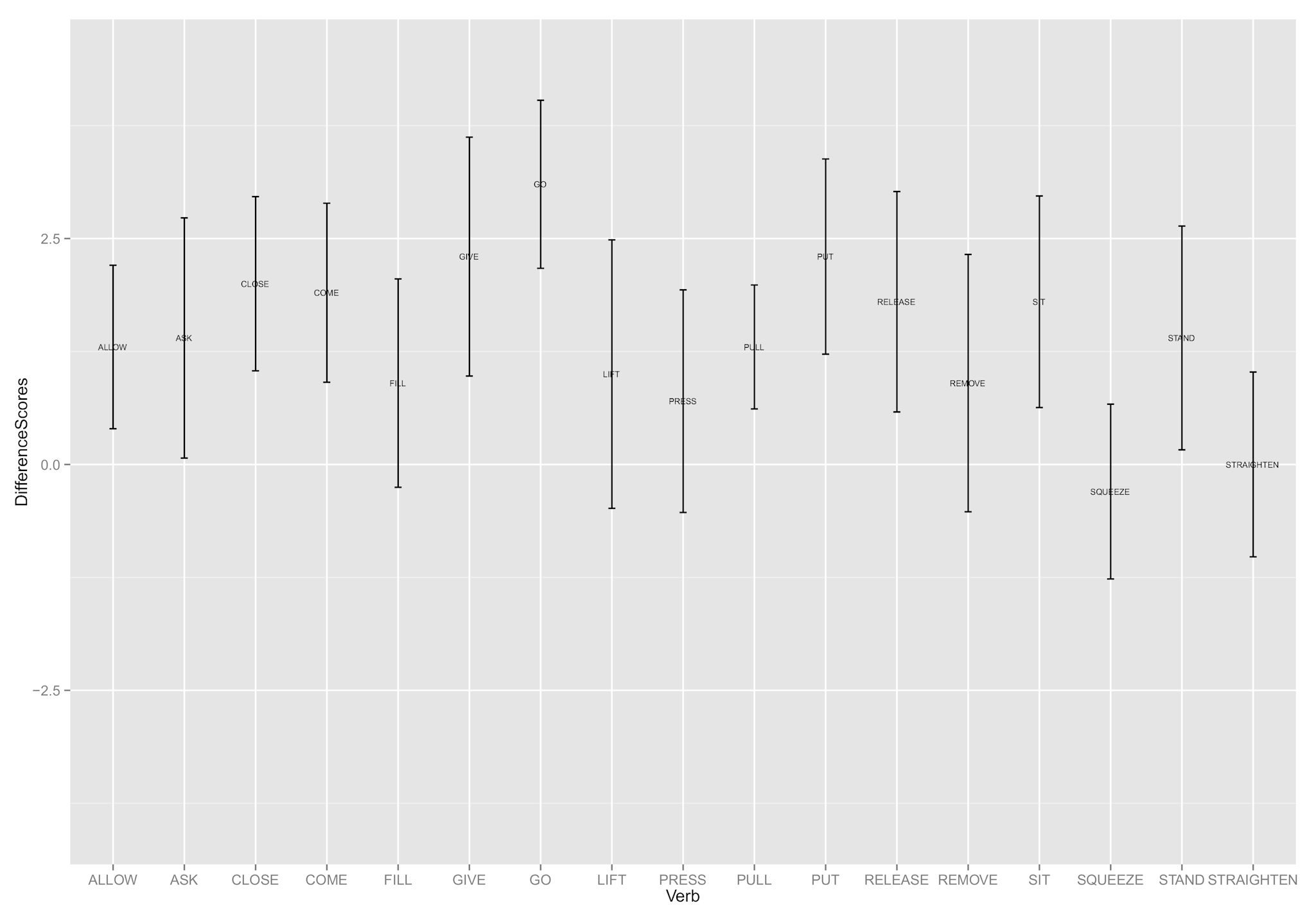

Supplement: Figure S2 — Mean Difference Scores for 5–6 Year Olds. Mean difference scores were calculated by subtracting the mean rating for each verb's un- form from the mean rating for each verb's bare form. If mean difference scores for verbs that do not take un- (i.e. “zero” verbs) fell below the value of zero then we assert that the child did not understand the meaning of the verb. Five-to-six year old children rated one “zero” verbs as more grammatical than its bare form equivalent (squeeze). Thus, we can be confident that test verbs used in the current study were suitable for use with this age-group. (TIF) [file pone.0110009.s002.tif]
