# Supplementary material for: Children Use Statistics and Semantics in the Retreat from Overgeneralization
Source: PLoS One. 2014 Oct 15;9(10):e110009. doi: 10.1371/journal.pone.0110009 (PMC4198212; doi:10.1371/journal.pone.0110009)
Supplement: Appendix S1 — Practice and Test Sentences for Production Study. (DOCX) [file pone.0110009.s004.docx]

**Appendix S1. Practice and Test Sentences for Production Study**

| **Practice Sentences (Production)** | |
| --- | --- |
| **Prime Sentences** | **Target Sentences** |
| Bart pinned the picture to the wall and then he unpinned it | Bart dressed the dog and then he… |
| Marge twisted the wire and then she untwisted it | Homer crossed his legs and then he… |
| Homer plugged in the toy and then he unplugged it | Lisa covered the ball and then she uncovered it |
|  |  |
| **Test Sentences (Production)** | |
| **Verb Set A** |  |
| **Prime Sentences** | **Target Sentences** |
| Bart chained the dog to a post and then he unchained it | **Bart embarrassed everyone and then he... |
| Bart laced his shoes and then he unlaced them | **Bart pulled the cord and then he... |
| Bart masked the cat and then he unmasked it | **Homer asked a question and then he... |
| Homer buckled his belt and then he unbuckled it | **Homer loosened his tie and then he... |
| Homer did his tie and then he undid it | **Homer stood on the box and then he... |
| Homer fastened his seatbelt and then he unfastened it | **Lisa believed in unicorns and then she... |
| Homer packed his case and then he unpacked it | **Lisa froze the ice lolly and then she... |
| Homer snapped the lego bricks together and then he unsnapped them | **Lisa opened the box and then she... |
| Homer wrapped the present and then he unwrapped it | **Lisa squeezed the sponge and then she... |
| Lisa bandaged her arm and then she unbandaged it | **Marge allowed Bart some chocolate and then she... |
| Lisa tied her shoelaces and then she untied them | **Marge closed the door and then she... |
| Marge deleted the email and then she undeleted it | **Marge released the bees and then she... |
| Bart clenched his fist and then he unclenched it | *Bart buttoned his shirt and then he... |
| Bart clogged the sink and then he unclogged it | *Bart hooked the picture on the wall and then he... |
| Bart linked the railway tracks and then he unlinked them | *Homer corked the bottle and then he... |
| Homer bolted the door and then he unbolted it | *Homer latched the gate and then he... |
| Homer clipped the papers together and then he unclipped them | *Homer veiled the bride and then he... |
| Homer tangled the strings and then he untangled them | *Lisa leashed the dog and then she... |
| Lisa braided her hair and then she unbraided it | *Lisa locked the door and then she... |
| Lisa curled her eyelashes and then she uncurled them | *Lisa rolled up the newspaper and then she... |
| Lisa strapped on her watch and then she unstrapped it | *Marge crumpled the paper and then she... |
| Marge coiled the rope and then she uncoiled it | *Marge reeled the cotton and then she... |
| Marge folded her arms and then she unfolded them | *Marge screwed the top on the container and then she... |
| Marge loaded the basket and then she unloaded it | *Marge zipped her coat and then she… |
| **Verb Set B** |  |
| **Prime Sentences** | **Target Sentences** |
| Bart buttoned his shirt and then he unbuttoned it | *Homer fastened his seatbelt and then he... |
| Bart hooked the picture on the wall and then unhooked it | *Lisa bandaged her arm and then she... |
| Homer corked the bottle and then he uncorked it | *Bart masked the cat and then he... |
| Homer latched the gate and then he unlatched it | *Homer wrapped the present and then he... |
| Homer veiled the bride and then he unveiled it | *Homer snapped the lego bricks together and then he... |
| Lisa leashed the dog and then she unleashed it | *Lisa tied her shoelaces and then she... |
| Lisa locked the door and then she unlocked it | *Bart laced his shoes and then he... |
| Lisa rolled up the newspaper and then she unrolled it | *Marge deleted the email and then she... |
| Marge crumpled the paper and then she uncrumpled it | *Homer buckled his belt and then he... |
| Marge reeled the cotton and then she unreeled it | *Homer did his tie and then he... |
| Marge screwed the top on the container and then she unscrewed it | *Bart chained the dog to a post and then he... |
| Marge zipped her coat and then she unzipped it | *Homer packed his case and then he... |
| Bart clenched his fist and then he unclinched it | **Homer came home and then he... |
| Bart clogged the sink and then he unclogged it | **Marge pressed the lever and then she... |
| Bart linked the railway tracks and then he unlinked them | **Bart filled the balloon and then he... |
| Homer bolted the door and then he unbolted it | **Homer sat on the dog and then he... |
| Homer clipped the papers together and then he unclipped them | **Homer tightened the screws and then he... |
| Homer tangled the strings and then he untangled them | **Marge put the book on the table and then she... |
| Lisa braided her hair and then she unbraided it | **Bart went to the hospital and then he... |
| Lisa curled her eyelashes and then she uncurled them | **Homer lifted his arms and then he... |
| Lisa strapped on her watch and then she unstrapped it | **Marge removed the television and then she... |
| Marge coiled the rope and then she uncoiled it | **Marge gave Bart a cookie and then she... |
| Marge folded her arms and then she unfolded them | **Homer bent the metal bar and then he... |
| Marge loaded the basket and then she unloaded it | **Marge straightened the picture and then she... |
|  | *= Target Sentence containing "un" verb |
|  | **= Target Sentence containing "zero" verb |
|  |  |
