# Supplementary material for: Children Use Statistics and Semantics in the Retreat from Overgeneralization
Source: PLoS One. 2014 Oct 15;9(10):e110009. doi: 10.1371/journal.pone.0110009 (PMC4198212; doi:10.1371/journal.pone.0110009)
Supplement: Appendix S2 — Practice and Test Sentences for Judgment Study. (DOCX) [file pone.0110009.s005.docx]

**Appendix S2. Practice and Test Sentences for Judgment Study**

| **Practice Sentences (Judgments)** | |
| --- | --- |
| 1) Lisa broke the cup (5/5) |  |
| 2) Lisa breakded the cup (1/5) |  |
| 3) Bart spilled soup onto his shirt (suggested score 4/5 as *spilt* is preferred in British English) | |
| 4) Homer eated the ice cream (suggested score 2/4 as more acceptable than *breakded*) | |
| 5) Homer drap water into the cup (suggested score 1/5 – over-irregularization of *drip*) | |
| 6) Bart sticked the stickers onto his shirt (suggested score 2-3/5; better than *breakded/drap*) | |
| 7) Lisa spreaded butter onto the bread (suggested score 4/5 as *spread* is the correct form, though many find *spreaded* acceptable) | |
|  |  |
| **Test Sentences (Judgments)** | |
| **Verb Set A** | **Verb Set B** |
| **Bart (un)embarrassed everyone | *Homer (un)fastened his seatbelt |
| **Bart (un)pulled the cord | *Lisa (un)bandaged her arm |
| **Homer (un)asked a question | *Bart (un)masked the cat |
| **Homer (un)loosened his tie | *Homer (un)wrapped the present |
| **Homer (un)stood on the box | *Homer (un)snapped the Lego bricks together/apart |
| **Lisa (un)believed in unicorns | *Lisa (un)tied her shoelaces |
| **Lisa (un)froze the ice lolly | *Bart (un)laced his shoes |
| **Lisa (un)opened the box | *Marge (un)deleted the email |
| **Lisa (un)squeezed the sponge | *Homer (un)buckled his belt |
| **Marge (un)allowed Bart some chocolate | *Homer (un)did his tie |
| **Marge (un)closed the door | *Bart (un)chained the dog to/from a post |
| **Marge (un)released the bees | *Homer (un)packed his case |
| *Bart (un)buttoned his shirt | **Homer (un)came home |
| *Bart (un)hooked the picture on/from the wall | **Marge (un)pressed the lever |
| *Homer (un)corked the bottle | **Bart (un)filled the balloon |
| *Homer (un)latched the gate | **Homer (un)sat on the dog |
| *Homer (un)veiled the bride | **Homer (un)tightened the screws |
| *Lisa (un)leashed the dog | **Marge (un)put the book on/off the table |
| *Lisa (un)locked the door | **Bart (un)went to the hospital |
| *Lisa (un)rolled (up) the newspaper | **Homer (un)lifted his arms |
| *Marge (un)crumpled the paper | **Marge (un)removed the television |
| *Marge (un)reeled the cotton | **Marge (un)gave Bart a cookie |
| *Marge (un)screwed the top onto/from the container | **Homer (un)bent the metal bar |
| *Marge (un)zipped her coat | **Marge (un)straightened the picture |
| *= Sentence containing "un" verb |  |
| **= Sentence containing "zero" verb |  |
