# Supplementary material for: Children Use Statistics and Semantics in the Retreat from Overgeneralization
Source: PLoS One. 2014 Oct 15;9(10):e110009. doi: 10.1371/journal.pone.0110009 (PMC4198212; doi:10.1371/journal.pone.0110009)
Supplement: Appendix S3 — CHILDES Frequency Counts of Each Verb (DOCX) [file pone.0110009.s006.docx]

**Appendix S3. CHILDES Frequency Counts of Each Verb.** Verbs in bold indicate that the verb has been registered less than 10 times in the CHILDES database. Also note that verbs which were heard by children were also produced at a similar frequency, indicating that spontaneous production of verbs were reflective of the frequency at which they were heard.

| **Verb** | **Input (1,678,227 utterances)** | **Children (854,696 utterances)** |  | **Verb** | **Input (1,678,227 utterances)** | **Children (854,696 utterances)** |
| --- | --- | --- | --- | --- | --- | --- |
| **bandage** | **2** | **2** |  | allow | 107 | 20 |
| buckle | 43 | 12 |  | ask | 2605 | 361 |
| button | 139 | 67 |  | believe | 530 | 112 |
| **chain** | **1** | **3** |  | bend | 235 | 75 |
| **cork** | **2** | **0** |  | close | 1693 | 982 |
| **crumple** | **2** | **1** |  | come | 31683 | 7205 |
| **delete** | **3** | **0** |  | **embarrass** | **6** | **0** |
| do | 59495 | 13776 |  | fill | 292 | 76 |
| fasten | 84 | 17 |  | freeze | 48 | 14 |
| hook | 161 | 41 |  | give | 10156 | 2572 |
| **lace** | **3** | **0** |  | go | 58170 | 23377 |
| **latch** | **7** | **0** |  | lift | 427 | 115 |
| **leash** | **0** | **0** |  | loosen | 27 | 13 |
| lock | 214 | 157 |  | open | 3709 | 2336 |
| **mask** | **2** | **3** |  | press | 492 | 185 |
| pack | 145 | 35 |  | pull | 2977 | 849 |
| **reel** | **0** | **0** |  | put | 38227 | 13281 |
| roll | 941 | 275 |  | **release** | **7** | **0** |
| screw | 243 | 119 |  | **remove** | **26** | **0** |
| snap | 177 | 70 |  | sit | 8349 | 3082 |
| tie | 685 | 367 |  | squeeze | 235 | 95 |
| **veil** | **0** | **0** |  | stand | 1858 | 768 |
| wrap | 242 | 81 |  | straighten | 85 | 11 |
| zip | 240 | 70 |  | tighten | 69 | 25 |
